# Metabolic syndrome and depression: evidence from a cross-sectional study of real-world data in Japan

**DOI:** 10.1265/ehpm.23-00369

**Published:** 2024-07-03

**Authors:** Kumi Sugimoto, Takuya Yamada, Atsushi Kitazawa, Yoshiharu Fukuda

**Affiliations:** Teikyo University Graduate School of Public Health, 2-11-1 Kaga, Itabashi-ku, Tokyo 173-8605, Japan

**Keywords:** Metabolic syndrome, Depression, Real-world data, Health claim data, Health checkup data, Cross-sectional study

## Abstract

**Background:**

Both metabolic syndrome (MetS) and depression are high priority health problems, especially for working age. Numerous studies have explored the link between metabolic syndrome and depression; however, not all of them have consistently demonstrated an association. The objective of this study was to determine whether there is an association between MetS and depression by analyzing extensive real-world data (RWD).

**Methods:**

Our data was drawn from insurance claims and health checkups of local government officials across all prefectures in Japan except for Tokyo in the 2019 fiscal year. According to the number of months with diagnosis of depression and prescription of antidepressants, the study participants were classified into the following categories: Certainly not Depression (CN), Possibly not Depression (PN), Possible Depression (PD), and Certain Depression (CD). Associations between MetS and its components—visceral obesity, hypertension, hyperlipidemia, and diabetes— and these categories of depression were analyzed by logistic regression.

**Results:**

The depression categories of the 130,059 participants were as follows: CN 85.2%; PN 6.9%; PD 3.9%; and CD 4.1%. For men, the adjusted odds ratio (AOR) for MetS were PN 0.94 (95% CI: 0.86–1.02), PD 1.31 (1.19–1.43), and CD 1.63 (1.50–1.76), with reference to CN. For women, AOR of MetS were PN 1.10 (0.91–1.32), PD 1.54 (1.24–1.91), and CD 2.24 (1.81–2.78). Among the MetS components, visceral obesity, hyperlipidemia, and diabetes were significantly associated with depression categories.

**Conclusions:**

In this study, we found a significant association between MetS and depression, this association being similar to that previously reported. Our findings provide robust evidence for linkage between MetS and depression, suggesting that analysis of RWD is useful for providing concrete evidence.

## Introduction

Metabolic syndrome (MetS) and depression are among the top priorities in public health. MetS comprises a cluster of metabolic abnormalities, including central obesity, hypertension, insulin resistance, and atherogenic dyslipidemia, and is associated with a strong risk of diabetes and cardiovascular disease (CVD) [1]. Depressive disorders are ranked globally as the single largest contributor to non-fatal loss of health and are also the major contributor to deaths by suicide [2].

There have been many previous studies on the relationship between MetS and depression. According to a systematic review, individuals with depression are at higher risk of MetS, with an odds ratio (OR) of 1.51 (95% confidence interval [CI] 1.36–1.68) for cross-sectional studies and 1.60 (95% CI 1.23–2.08) for cohort studies [5]. Another systematic review reported an OR of 1.42 (95% CI 1.28–1.57) for cross-sectional studies [6]. To our knowledge, there have been five Japanese studies on the relationship between MetS and depression [7–11]. The relationship between MetS and depression is inconsistent across these studies, ranging from no relationship [9] to an adjusted odds ratio (AOR) of more than three [7]. Each study determined depression differently, some using a questionnaire [8–10], one by interviews to physicians [7], and another by prescribing antidepressants [11]. Furthermore, most of these studies were sampling surveys and were therefore limited regarding the number of study participants and their representativeness [7–10].

The association between MetS and depression might differ depending on region and country. The prevalence of MetS in Japan is 28.2% for men and 10.3% for women [12]. The prevalence of MetS and obesity in East Asian countries including Japan is extremely low compared to Western countries [13, 14]. On the other hand, depression is influenced by social and cultural aspects, and the prevalence varies by region and country [15]. Therefore, exploring the association between MetS and depression in each region and country will provide important insights for the mechanisms and countermeasures from an international perspective.

The evidence of association of MetS and depression implies a high likelihood that these conditions co-occur. This comorbidity will greatly increase the risk of morbidity and mortality. Thus, much attention is being given to preventing MetS and depression. In Japan, specific health checkups and health guidance for MetS are being implemented [3]. Further, various measures for addressing depression have been implemented, including prevention of overwork by individuals of working age [4]. However, these measures are not well coordinated, not considering the synergistic risks of MetS and depression.

Recently, there has been growing utilization of real-world data (RWD) [16–18]. RWD refers to large-scale data collected mainly from actual medical records and health checkups. In Japan, public medical insurers possess extensive data on insurance claims and health checkups and there is a concerted effort to promote the use of RWD in disease prevention and management of healthcare costs, commonly referred to as “data-health” [19]. Because RWD encompasses these medical care and health checkup data, it has significant potential for facilitating more precise examination of the association between MetS and depression.

Thus, we hypothesized that MetS and depression are associated in Japanese population as well, and that a more reliable relationship can be revealed by using a large-scale data. Under this hypothesis, the aim of the present study was to examine the association between MetS and depression using RWD (insurance claims and health checkups) among Japanese individuals and to discuss the validity and effectiveness of RWD analysis.

## Methods

### Study population and data

This was a cross-sectional study using data from insurance claims and health checkups during the 2019 fiscal year. Data were obtained from a health insurance association that enrolls local government officials of all prefectures (N = 46) in Japan other than Tokyo. The receipts for insurance claims list the primary diagnosis and information regarding all prescriptions for drugs. The data from health checkups include all items of specific health checkups (“Tokutei Kenshin”) [3].

We used the data of subjects aged from 40 to 74 years, this age group being eligible for specific health checkups (n = 253,592). Exclusion criteria were: (1) not having undergone health checkups; (2) aged 65 years or older; (3) not insured, such as a spouse and family members; (4) missing or outlier data concerning MetS; and (5) missing health behavior items used in the analysis. The outliers for laboratory data were: waist circumference (WC) <30 or ≥200 cm, systolic blood pressure (SBP) <50 or ≥250 mmHg, diastolic blood pressure (DBP) <30 or ≥150 mmHg, triglycerides (TG) <20 or ≥2000 mg/dL, high-density lipoprotein cholesterol (HDL-C) <10 or ≥200 mg/dL, fasting blood glucose (FBG) <30 mg/dL. The criteria of outliers were based on previous studies [20, 21], to exclude the subjects whose data were obviously incorrect due to such as entry errors.

**Fig. 1:**
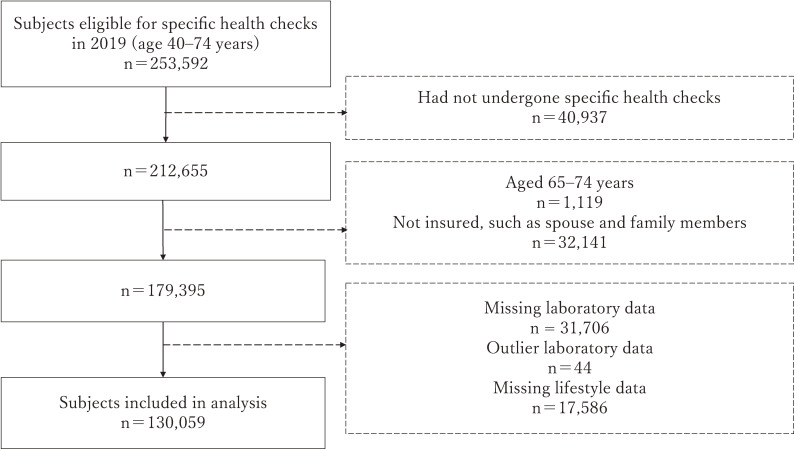
Flowchart of selection of study participants

Figure 1 shows the participant selection flow. After applying these criteria, 130,059 participants were included in the analysis. Most of missing laboratory data (n = 31,387) were found in FBG, since occasional blood sugar and/or hemoglobin A1c (HbA1c) were measured instead of FBG.

### Definition of MetS and its components

We categorized those who were matched the definition of MetS in the guideline of the Japanese Society of International Medicine as MetS [22]. This definition is visceral obesity (WC ≥85 cm for men and ≥90 cm for women) and at least two of the following three metabolic components: hypertension (SBP ≥130 mmHg and/or DBP ≥85 mmHg), hyperlipidemia (TG ≥150 mg/dL and/or HDL-C <40 mg/dL), and hyperglycemia (FBG ≥110 mg/dL).

### Categories of depression

Because receipts for insurance claims in Japan list multiple injuries and illnesses, they do not enable precise identification of depression unless it is the primary diagnosis [23]. Further, antidepressant and depression-related (neuropsychiatric) drugs may be prescribed for conditions other than depression. We therefore classified participants on the basis of a combination of number of months of receipts with a diagnosis of depression and prescription of antidepressants during the 12 months studied. For diagnosis, we counted months with receipts naming mood disorders, including manic-depressive disorders and stress-related and somatoform disorders (F30-49 in ICD-10). For prescriptions, we counted the months in which hypnotic sedatives, anxiolytics and psychoneurotic drugs were prescribed (112 and 117 in the drug classification codes) [24]. Then, using the matrix shown in Table 1, the following diagnostic categories were allocated: Certainly Not Depression (CN), Possibly Not Depression (PN), Possible Depression (PD), and Certain Depression (CD).

**Table 1:** Categorization of depression

	**Number of months with prescription of anti-depression drug**		

**Number of months with diagnosis ** **of depressive disorder**	**0**	**1 to 5**	**≥6**
0	CN (n = 110,757)	PN (n = 7,675)	PD (n = 2,351)
1 to 5	PN (n = 991)	PD (n = 2,353)	CD (n = 267)
≥6	PN (n = 244)	PD (n = 362)	CD(n = 5,059)

CN = Certainly Not Depression, PN = Possibly Not Depression, PD = Possible Depression, CD = Certain Depression

### Covariates

We treated age and health behaviors as possible confounders. For health behaviors, current smoking, alcohol consumption, physical inactivity, and insufficient sleep were selected from the standard questionnaire items of the specific health checkups [25].

We used the following definitions: current smoking = have smoked more than 100 cigarettes in total or have smoked for more than 6 months and have smoked in the last month; alcohol consumption = drink alcohol daily or occasionally; physical inactivity = have not engaged in light, sweat-inducing exercise for more than 30 minutes at a time, at least 2 days a week, for more than 1 year; and insufficient sleep = not feeling rested after sleep.

### Analysis

First, we compared the prevalence of MetS and its components and health behaviors in the four depression categories. Next, we performed logistic regression analysis with MetS or its components as the objective variables and depression category and covariates as explanatory variables. We then estimated OR and its 95% confidence intervals (95% CI). Model 1 estimated crude OR (COR) and Model 2 estimated AOR for all explanatory variables. All analyses were conducted by sex.

As a sensitivity analysis, logistic regression analysis was performed on 179,395 individuals, including those with missing health behaviors, to estimate COR for the four categories of depression.

IBM SPSS Statistics version 28.0 was used for the analysis. The significance level was set at less than 0.05 bilaterally.

### Ethical considerations

This study was conducted after obtaining approval from the Teikyo University Ethics Review Committee (Teirin 22-073, approval date: August 17th, 2022).

## Results

Table 1 shows the number of persons in the depression categories derived from the matrix of months of depression diagnosis and antidepressant prescriptions: 110,757 (85.2%) for CN, 8,910 (6.9%) for PN, 5,066 (3.9%) for PD and 5,326 (4.1%) for CD.

Table 2 is a summary of characteristics of study participants including depression category, MetS and its components, and health behaviors by sex. The proportion of men was 70.3%, and the age did not differ significantly between sexes. The proportions of CD, MetS and its components, and most unhealthy behaviors were higher in men.

**Table 2:** Characteristics of the analyzed participants

	**Male ** **(n = 91,454)**		**Female ** **(n = 38,605)**	
Age in years [mean (SD)]	51.4	(6.0)	49.1	(5.8)
Depression category^a^ [n (%)]				
CN	77,999	(85.3%)	32,758	(84.9%)
PN	5,813	(6.4%)	3,097	(8.0%)
PD	3,558	(3.9%)	1,508	(3.9%)
CD	4,084	(4.5%)	1,242	(3.2%)

Metabolic syndrome [n (%)]	11,959	(13.1%)	1,050	(2.7%)
Visceral obesity [n (%)]	40,236	(44.0%)	5,205	(13.5%)
Hypertension [n (%)]	32,512	(35.6%)	6,682	(17.3%)
Dyslipidemia [n (%)]	22,455	(24.6%)	2,826	(7.3%)
Hyperglycemia [n (%)]	14,291	(15.6%)	2,069	(5.4%)

Smoking^b^ [n (%)]	17,223	(18.8%)	1,257	(3.3%)
Alcohol consumption^c^ [n (%)]	66,368	(72.6%)	19,288	(50.0%)
Physical inactivity^d^ [n (%)]	65,849	(72.0%)	33,725	(87.4%)
Insufficient sleep^e^ [n (%)]	58,201	(63.6%)	19,636	(50.9%)

^a^CN = Certain Not Depression, PN = Possible Not Depression, PD = Possible Depression CD = Certain Depression.

^b^Have smoked more than 100 cigarettes in total or for more than 6 months and have smoked in the last month.

^c^Drink alcohol daily or occasionally.

^d^Have not done light, sweat-inducing exercise for more than 30 minutes at a time, at least 2 days a week, for more than 1 year.

^e^Not feeling rested after sleep.

Table 3 shows the association of MetS with depression categories and other variables for men. The association with depression categories increased in a dose–response manner: AORs compared with CN were 0.93 (95% CI, 0.86–1.01) for PN, 1.30 (95% CI, 1.19–1.43) for PD, and 1.61 (95% CI, 1.49–1.75) for CN. Smoking and physical inactivity were significantly and positively associated with MetS, whereas drinking alcohol and insufficient sleep were not significantly or modestly associated.

**Table 3:** The association of metabolic syndrome (MetS) with depression categories and other variables for males

	**Prevalence ** **of MetS**	**Model 1 (Crude)**				**Model 1 (Adjusted)**			
	
		**OR**	**95% CI**		**p-value**	**AOR^a^**	**95% CI**		**p-value**
**Depression categories^b^**									
CN	12.6%	1.00	Reference			1.00	Reference		
PN	12.3%	0.97	0.89	1.05	0.402	0.93	0.86	1.01	0.081
PD	16.6%	1.37	1.25	1.50	<0.001	1.30	1.19	1.43	<0.001
CD	19.5%	1.67	1.54	1.81	<0.001	1.61	1.49	1.75	<0.001
**Age (1-year increase)**		1.04	1.04	1.05	<0.001	1.05	1.04	1.05	<0.001
**Smoking**									
No	12.2%	1.00	Reference			1.00	Reference		
Yes	17.0%	1.47	1.41	1.54	<0.001	1.43	1.37	1.50	<0.001
**Alcohol consumption**									
No	13.3%	1.00	Reference			1.00	Reference		
Yes	13.0%	0.97	0.93	1.01	0.155	0.98	0.94	1.03	0.448
**Physical inactivity**									
No	10.5%	1.00	Reference			1.00	Reference		
Yes	14.1%	1.40	1.34	1.47	<0.001	1.39	1.33	1.46	<0.001
**Insufficient sleep**									
No	13.7%	1.00	Reference			1.00	Reference		
Yes	12.7%	0.91	0.88	0.95	<0.001	0.95	0.91	0.99	0.016

AOR = adjusted odds ratio, CI = confidence interval, OR = odds ratio

^a^Adjusted for age, smoking, alcohol consumption, physical inactivity, and insufficient sleep.

^b^CD = Certain Depression, CN = Certainly Not Depression, PD = Possible Depression, PN = Possibly Not Depression.

The results for women are shown in Table 4. The associations of MetS were similar to those for men; however, they were stronger than for men: the AORs were 1.41 (95% CI, 1.07–1.85) for PD and 2.09 (95% CI, 1.61–2.70) for CN. The association with smoking was stronger than in men, whereas the association with physical inactivity was weaker. Drinking alcohol and insufficient sleep were not significantly or modestly associated with MetS.

**Table 4:** The association of metabolic syndrome (MetS) with depression categories and other variables for females

	**Prevalence of MetS**	**Model 1 (Crude)**				**Model 1 (Adjusted)**			
	
		**OR**	**95% CI**		**p-value**	**AOR^a^**	**95% CI**		**p-value**
**Depression^b^**									
CN	2.5%	1.00	Reference			1.00	Reference		
PN	3.0%	1.20	0.97	1.49	0.095	1.12	0.90	1.39	0.301
PD	3.8%	1.54	1.17	2.02	0.002	1.41	1.07	1.85	0.014
CD	5.4%	2.19	1.70	2.83	<0.001	2.09	1.61	2.70	<0.001
**Age (1-year increase)**		1.07	1.05	1.08	<0.001	1.07	1.06	1.08	<0.001
**Smoking**									
No	2.6%	1.00	Reference			1.00	Reference		
Yes	5.0%	1.94	1.50	2.52	<0.001	1.96	1.51	2.55	<0.001
**Alcohol consumption**									
No	2.9%	1.00	Reference			1.00	Reference		
Yes	2.5%	0.87	0.77	0.98	0.026	0.90	0.79	1.01	0.080
**Physical inactivity**									
No	2.3%	1.00	Reference			1.00	Reference		
Yes	2.8%	1.24	1.02	1.52	0.033	1.32	1.08	1.62	0.007
**Insufficient sleep**									
No	3.0%	1.00	Reference			1.00	Reference		
Yes	2.5%	0.83	0.73	0.93	0.002	0.88	0.78	0.99	0.039

AOR = adjusted odds ratio, CI = confidence interval, OR = odds ratio

^a^Adjusted for age, smoking, alcohol consumption, physical inactivity, and insufficient sleep.

^b^CD = Certain Depression, CN = Certainly Not Depression, PD = Possible Depression, PN = Possibly Not Depression.

Table 5 and Table 6 shows the associations between MetS components and depression categories. For both men and women, we found significant associations for visceral obesity, dyslipidemia, and hyperglycemia, but not for hypertension. The associations were stronger in women, especially for dyslipidemia.

**Table 5:** The association of components of metabolic syndrome with depression categories for males

**Depression^a^**	**Visceral obesity**					**Hypertension**				
	
	**Prevalence**	**AOR^b^**	**95% CI**		**p-value**	**Prevalence**	**AOR^b^**	**95% CI**		**p-value**
CN	43.2%	1.00	Reference			35.6%	1.00	Reference		
PN	43.7%	1.00	0.94	1.05	0.900	35.2%	0.94	0.89	0.99	0.025
PD	48.7%	1.19	1.12	1.28	<0.001	37.0%	1.07	0.99	1.14	0.082
CD	54.8%	1.53	1.43	1.63	<0.001	33.4%	0.96	0.90	1.02	0.207

**Depression^a^**	**Dyslipidemia**					**Hyperglycemia**				
	
	**Prevalence**	**AOR^b^**	**95% CI**		**p-value**	**Prevalence**	**AOR^b^**	**95% CI**		**p-value**

CN	23.9%	1.00	Reference			15.2%	1.00	Reference		
PN	22.9%	0.96	0.90	1.02	0.180	15.2%	0.91	0.84	0.98	0.015
PD	29.3%	1.29	1.19	1.39	<0.001	19.9%	1.30	1.20	1.42	<0.001
CD	34.9%	1.65	1.54	1.76	<0.001	20.1%	1.40	1.29	1.52	<0.001

AOR = adjusted odds ratio, CI = confidence interval

^a^CD = Certain Depression, CN = Certainly Not Depression, PD = Possible Depression, PN = Possibly Not Depression.

^b^Estimated by logistic regression with each component of metabolic syndrome as the objective variable and depression categories, age, smoking, alcohol consumption, physical inactivity, and insufficient sleep as explanatory variables.

**Table 6:** The association of between components of metabolic syndrome with depression and for females

**Depression^a^**	**Visceral obesity**					**Hypertension**				
	
	**Prevalence**	**AOR^b^**	**95% CI**		**p-value**	**Prevalence**	**AOR^b^**	**95% CI**		**p-value**
CN	13.0%	1.00	Reference			17.2%	1.00	Reference		
PN	14.0%	1.03	0.92	1.14	0.651	17.8%	0.98	0.89	1.08	0.730
PD	17.4%	1.32	1.15	1.52	<0.001	18.2%	1.03	0.90	1.17	0.717
CD	20.9%	1.71	1.49	1.98	<0.001	16.9%	0.99	0.85	1.15	0.876

**Depression^a^**	**Dyslipidemia**					**Hyperglycemia**				
	
	**Prevalence**	**AOR^b^**	**95% CI**		**p-value**	**Prevalence**	**AOR^b^**	**95% CI**		**p-value**

CN	6.9%	1.00	Reference			5.2%	1.00	Reference		
PN	7.8%	1.10	0.96	1.26	0.188	5.8%	1.02	0.87	1.20	0.809
PD	9.9%	1.41	1.18	1.68	<0.001	7.0%	1.26	1.02	1.55	0.031
CD	13.6%	2.05	1.73	2.43	<0.001	7.4%	1.47	1.18	1.83	<0.001

AOR = adjusted odds ratio, CI = confidence interval, OR = odds ratio

^a^CD = Certain Depression, CN = Certainly Not Depression, PD = Possible Depression, PN = Possibly Not Depression.

^b^Estimated by logistic regression with each component of metabolic syndrome as the objective variable and depression categories, age, smoking, alcohol consumption, physical inactivity, and insufficient sleep as explanatory variables.

In the analysis of participants with missing values performed as a result of the sensitivity analysis, the CORs of MetS compared with CN in men were 0.94 (95% CI, 0.86–1.02) for PN, 1.31 (95% CI, 1.19–1.43) for PD, and 1.63 (95% CI, 1.50–1.76) for CD. CORs in women were 1.10 (95% CI, 0.91–1.32) for PN, 1.54 (95% CI, 1.24–1.91) for PD, and 2.24 (95% CI, 1.81–2.78) for CD. These results are similar to those of the subjects without missing values.

## Discussion

The present study using RWD (data from insurance claims and health checkups) revealed a significant association between MetS and depression in a population of working age in Japan. The study has several strengths compared with previous Japanese studies [7–11]. Additionally, the association we identified is consistent with the results of systematic reviews of international studies [6, 26]. Therefore, our study provides definite, robust evidence of an association between MetS and depression in Japan. Our results demonstrate the validity and usefulness of RWD, such as medical records and health checkup data.

The present study has several strengths. Our large sample size enabled us to minimize potential biases. The high rate of participation in health checkups (84%) and the fact that insurance claims were available for the entire study population would have eliminated selection bias. Using medical records to identify depression would have minimized information (diagnostic) bias compared with other methods such the self-reporting and questionnaires used in previous studies [7–10]. Local government officials, who were the focus of our analysis, exhibit low turnover rates that are attributable to illness, this in turn being attributable to the ample benefits and support systems provided in their workplaces. This noteworthy characteristic contributed to mitigation of the healthy worker effect [27, 28]. These strengths allowed us to accurately identify an association between MetS and depression.

The results of the four previous studies in Japan are inconsistent. Two studies by Takeuchi et al. found a significant association between MetS and depression with AORs of 3.8 [7] and 1.6 [7, 10]. Nishina et al. found that the association between MetS and depression scores was significant for men but not for women [8]. Kimura et al.’s study did not show a significant association between MetS and depression [9]. These differences are possibly attributable to differences in study populations and methods of analysis. In particular, these studies had small populations (458 to 1,613). Considering the strengths of the present study, we have provided concrete evidence of an association between MetS and depression in Japan.

The strength of association in the present study is comparable to that reported by previous international studies. The ORs of meta-analyses are 1.51 for cross-sectional studies and 1.6 for cohort studies [5]. Generally, epidemiological relationships differ by country, race, and other factors. The OR in a nationally representative survey in South Korea was 1.41 [29], which is similar to that found in the present study. Therefore, we conclude from our findings using RWD that the association between MetS and depression in Japan is comparable to that found in other industrial countries.

The association with depression varies by MetS components. In the present study, we found significant associations for visceral obesity, dyslipidemia, and hyperglycemia, but not for hypertension. This is inconsistent with some of the findings of previous studies [30, 31]. In most studies, visceral obesity (abdominal circumference) and dyslipidemia were found to be significantly and strongly associated [32, 33]. In contrast, hyperglycemia (blood glucose) and hypertension (blood pressure) were reportedly weakly or not significantly associated with depression [32]. The associations between diabetes and hypertension and depression have been examined separately. Meta-analyses have concluded that depression increases the risk of diabetes and conversely, that diabetes increases the risk of depression [34]. The OR of the association found by this meta-analysis was 1.41 [34], which is similar to our findings (1.40 for men and 1.47 for women). A meta-analysis on whether depression increases the risk of hypertension has shown a significant, but weak, relationship [31]. Thus, the results of the present study are in line with those of previous studies.

There are three main hypotheses for the association between MetS and depression. The first is related to health behaviors. Physical inactivity and overeating, both of which are common in individuals with depression, pose significant risks for development of MetS [35]. Furthermore, even when health guidance is provided, individuals with depression may face challenges in adhering to such guidance because of the nature of their condition. The second hypothesis is based on the fact that certain antidepressants are known to cause obesity and metabolic dysregulation [36]. The third hypothesis is based on biological mechanisms as factors in the association. As the biological mechanism, Penninx and Lange summarized that a multitude of genetic vulnerabilities and pathophysiological mechanisms (causing central and peripheral activation of immuno-metabolic and endocrine systems) have been implicated in the development of MetS and psychiatric disorders such as depression [36]. Given that the present study was a cross-sectional study, we could not determine which of these factors are significant. However, the use of longitudinal and detailed RWD, including medications and health behaviors, may help to elucidate these hypotheses, especially the effects of medications and health behaviors.

Concerning risk behaviors, we found conventionally positive association between MetS and smoking and physical inactivity. While insufficient sleep was negatively associated with MetS. Previous studies about MetS and sleep duration failed to find consistence associations [37, 38]. The question related sleep in our study was one item about resting after sleep, and thus it is difficult to assess quality and quality of sleep, unlike established questionnaires [39]. Also, out result might imply the contrary cause-effect relationship that those who have risk factors including MetS are likely to take care of their sleep. This contrary cause-effect relationship could be applied to the negative association between MetS and alcohol consumption for females.

This study has several limitations that should be acknowledged. The first limitation pertains to the cross-sectional design, which precluded establishing a clear causal relationship. However, the objective of this study was to examine the association between MetS and depression rather than to establish causality. The findings of a systematic review of longitudinal studies of obesity and depression suggested that they have a bidirectional causal relationship [25]. Similarly, MetS and depression are expected to have a bidirectional causal relationship. There is one longitudinal study in Japan, showing the significant association between MetS and incident of depression [11]. The association of the study was similar pattern of, but weaker than the results of our study. The stronger association of our cross-sectional study implies that the bidirectional causality and comorbidity are important for prevention and treatment of MetS and depression.

The second limitation is the uncertainty of diagnoses and other information in health claims [22]. This uncertainty likely resulted in some misclassification. In an attempt to address this limitation, we classified the study participants into four categories based on two aspects: depression diagnosis and antidepressant prescriptions. In comparison with previous studies, in which one aspect such as a questionnaire and prescribing antidepressants were used [7–11], the present study possibly reduced a likelihood of the misclassification and identified a dose–response relationship.

The third limitation concerns the representativeness of the study sample. Tokyo prefecture, which is the largest prefecture, was excluded, since the prefecture has a unique health insurance. However, our data included some prefectures cover metropolitan areas such as Osaka, Nagoya and Fukuoka. Therefore, the influence of exclusion of Tokyo might be small. In addition, it is important to note that our data were from local government officials across Japan, which, while not specific, can be considered relatively representative of the Japanese population. It would be difficult to identify alternative data sources that are both nationally representative and would provide a larger sample than we had.

Finally, this study has several noteworthy implications. Firstly, our clarification of the association between MetS and depression suggests the importance of prevention of both conditions. Programs for preventing non-communicable or lifestyle-related diseases sometimes exclude patients with mental disorders such as depression [40, 41]. However, our clarification of the link between MetS and depression points to a growing need for more comprehensive prevention and disease management strategies. Secondly, RWD are useful both for elucidating the association between MetS and depression and for shedding light on the connections between various other diseases. The employment of larger-scale and longitudinal data enables accumulation of more substantial evidence than is possible with traditional epidemiological studies.

In conclusion, we investigated the association between MetS and depression using data from numerous Japanese health checkups and insurance claims and found a significant association between MetS and depression. Our findings imply that careful consideration and control of metabolic risk factors for people with depression, while prevention of depression for people with metabolic risks is required. The strength of the association was broadly consistent with that found by previous international studies. Using the data from large samples of local government officials across the country, our result is generalized for Japanese population more than other studies using specified populations. Therefore, the present study provides robust evidence for the Japanese population, stronger evidence for an association between MetS and depression, and the validity of RWD analysis.

## Abbreviations

AOR: adjusted odds ratio
CD: certain depression
CI: confidence interval
CN: certainly not depression
COR: crude odds ratio
DBP: diastolic blood pressure
FBG: fasting blood glucose
HDL-C: high-density lipoprotein cholesterol
MetS: metabolic syndrome
OR: odds ratio
PD: possible depression
PN: possibly not depression
SBP: systolic blood pressure
TG: triglycerides
RWD: real-world data
WC: waist circumference

## Declarations

### Availability of data and material

Data cannot be shared for privacy or ethical reasons.
